# Neuroimmune activation in temporal lobe epilepsy patients with worsening seizure following the COVID-19 pandemic: A [^18^F]DPA-714 PET/MR study

**DOI:** 10.1126/sciadv.adu5874

**Published:** 2026-01-30

**Authors:** Ling Xiao, Li Qin, Tao Jiang, Ming Qu, Manliu Hou, Yongxiang Tang, Shuo Hu, Li Feng

**Affiliations:** ^1^ Department of Nuclear Medicine, Xiangya Hospital, Central South University, Changsha, Hunan, China.; ^2^ Department of Neurology, Xiangya Hospital, Central South University, Changsha, Hunan, China.; ^3^ National Clinical Research Center for Geriatric Diseases, Xiangya Hospital, Central South University, Changsha, Hunan, China.; ^4^ Key Laboratory of Biological Nanotechnology of National Health Commission, Xiangya Hospital, Central South University, Changsha, Hunan, China.

## Abstract

Patients with temporal lobe epilepsy (TLE) frequently experience worsening epilepsy following COVID-19, referred to as post–COVID-19 active TLE. While neuroinflammatory changes are suspected in these patients, measurements of both central and systemic inflammation in the brain remain unexplored. We investigate whether the translocator protein standardized uptake value ratio (TSPO SUVr), a quantifiable marker of neuroinflammation using positron emission tomography (PET), is elevated in the brains of patients with post–COVID-19 active TLE. In addition, we examine correlations between TSPO SUVr and inflammatory factors to identify potential peripheral blood inflammatory predictors of post–COVID-19 active epilepsy. Our study highlights the presence of widespread neuroinflammation in the brain and increased levels of inflammatory cytokines in the plasma of individuals with post–COVID-19 active TLE. Furthermore, strong correlations between plasma levels of interleukin-1β (IL-1β), IL-10, and interferon-γ (IFN-γ) and neuroimmune activation suggest the potential for integrating plasma inflammatory factors with TSPO PET as a dependable approach for clinical diagnosis, dynamic monitoring, and assessment of immune-based therapeutic efficacy in TLE-associated neuroinflammation.

## Teaser

TSPO PET and inflammatory markers reveal spread neuroinflammation in temporal lobe epilepsy with seizure worsening after COVID-19.

## INTRODUCTION

The COVID-19 pandemic has resulted in a global public health crisis (*1*), with acute COVID-19 infections affecting nearly one-third of the population in affected countries (*2*), and nearly every individual in China experiencing at least one acute episode of the disease (*3*). Neurological symptoms, reported in up to 21.3% of patients with COVID-19 (*4*), frequently include exacerbations of epilepsy, classified as post–COVID-19 active epilepsy (*5*–*7*). In addition, retrospective studies have shown that the risk of developing epilepsy remains elevated at the end of a 2-year follow-up period (*8*), making post–COVID-19 active epilepsy a notable health problem.

Neuroinflammation is known to contribute to temporal lobe epilepsy (TLE) (*9*). Animal studies demonstrate that neuroinflammation plays an important role in pathophysiological processes (*10*–*13*). Many inflammatory factors including tumor necrosis factor–α (TNF-α) and interleukins (ILs) are up-regulated in animal models of epilepsy, and their activation correlates with seizure frequency (*14*, *15*). Seizures can cause neuronal injury, resulting in the release of adenosine triphosphate, glutamate, and reactive oxygen species. These molecules are recognized by microglia, triggering their activation and subsequent release of proinflammatory mediators, including IL-1β, IL-6, and TNF-α. These compounds initiate specific inflammatory signaling cascades that worsen epileptic injury (*16*). Human studies have also observed increased expression of proinflammatory markers in neurosurgical samples obtained from individuals with TLE (*17*), with enriched neuroinflammatory markers found in epileptogenic focal regions corroborated by translocator protein 18 kDa (TSPO) positron emission tomography (PET) in vivo imaging (*18*, *19*).

Recently, an increasing number of studies have reported cases of worsening epilepsy following COVID-19 (*5*, *20*), indicating a close association between neuroinflammation after COVID-19 infection and the development of epilepsy. Various events occurring during the acute phase of COVID-19, such as elevation of peripheral cytokine signaling and inflammatory responses in the brain (*21*), the emergence of new hypoxic or vasoocclusive lesions, and possible direct viral invasion into the brain, can provoke a central immune response (*22*). Microglia, functioning as intracerebral macrophages, act as key players in this response. In the context of neocoronavirus involvement in the central nervous system (CNS), microglia can transition from a steady state to an activated state, triggering a neuroinflammatory cascade and elevating TSPO expression. This change is particularly evident in the epileptogenic focus of patients with epilepsy following COVID-19, where substantial aggregation of activated microglia has been observed (*23*–*25*). However, while autopsy studies have identified microglial cells or astroglial nodules within the epileptogenic focus of patients with TLE who succumbed to severe or critical COVID-19, these findings are constrained by the predominance of samples from more severely affected individuals. Thus, it is challenging to fully elucidate the actual status of patients with mild to moderate COVID-19 (*26*). Consequently, further investigations are needed to determine whether the morphology, function, and spatiotemporal environment of microglia are altered in the brains of patients with TLE following COVID-19.

TSPO PET imaging is a strategy to detect microglial activation in vivo. TSPO expression is relatively low under physiologic conditions but becomes markedly up-regulated in response to various inflammation-related diseases, thereby making it a potential biomarker for monitoring inflammatory disease progression (*27*). PET imaging using TSPO radioligands has been widely used to visualize microglial activation in conditions such as traumatic brain injury (*28*), Alzheimer’s disease (*29*), autoimmune encephalitis (*27*), and persistent depressive and/or cognitive symptoms after COVID-19 (*2*). However, whether neuroinflammatory responses occur in post–COVID-19 epilepsy and whether such responses contribute to increased seizure severity or disease progression remain unclear and require further investigation.

The study aims to compare neuroinflammation across the entire brain in patients with TLE experiencing worsening seizures post–COVID-19 with those whose seizures have not worsened, using integrated synchronized PET/magnetic resonance (MR) imaging with the second-generation TSPO radioligand [^18^F]DPA-714. The primary objective is to elucidate the pathogenesis of active epilepsy after COVID-19 and to provide a clinical rationale for developing therapeutic strategies targeting inflammation interventions. Furthermore, the study will explore the intrinsic relationship between TSPO standardized uptake value ratio (SUVr) and various inflammatory factors to identify potential peripheral blood inflammatory predictors of post–COVID-19 active epilepsy, facilitating the early identification of neuroinflammation for effective intervention in TLE.

## RESULTS

### Participant characteristics

In total, 45 participants were recruited in this study, comprising 17 individuals with post–COVID-19 active TLE, 17 individuals with post–COVID-19 nonactive TLE, and 11 healthy controls (HCs). The demographic and clinical characteristics of participants are summarized in Table 1 and table S1. There were no significant differences in age, sex, and years of education in the study between patients with TLE and HCs (*P* > 0.05). In addition, no significant differences were found between the post–COVID-19 active TLE group and the post–COVID-19 nonactive TLE group in terms of seizure frequency or the number of medications taken (*P* > 0.05). However, the age at seizure onset was older in the post–COVID-19 nonactive TLE group compared to the active group (*P* = 0.0049). Among patients with post–COVID-19 active TLE, five had cognitive impairment, two experienced depression, and one suffered from anxiety. In contrast, three patients in the post–COVID-19 nonactive TLE group had cognitive impairment. Fifteen out of the patients with active TLE after COVID-19 were vaccinated, with one completing two shots of the vaccine and 14 completing three shots, while two remained unvaccinated. Among patients with nonactive post–COVID-19 TLE, 16 were vaccinated, with one completing two shots of the vaccine and 15 completing three shots, leaving only one remaining unvaccinated. All 11 HCs completed three shots of vaccination.

**Table 1.** Demographic and clinical characteristics of study participants. Data are means (SD). *P* values are for one-way ANOVA (continuous variables) or χ2 (categorical variables). Statistical significance between groups is indicated by bold styling and asterisk (*P < 0.05).

	Post–COVID-19 active TLE (*n* = 17)	Post–COVID-19 nonactive TLE (*n* = 17)	Healthy control (*n* = 11)	*P* value	Contrasts	*P* value
Sex (female/male)	6/11	10/7	7/4	0.2469	-	-
Age (years)	40.41 (12.40)	31.94 (6.99)	36.19 (11.21)	0.1002	-	-
Education (years)	11.29 (2.85)	12.88 (2.62)	12.27 (5.39)	0.3072	-	-
Age at seizure onset (years)	31.53 (12.90)	20.41 (7.98)	-	-	Active TLE group > nonactive TLE group	0.0049*
Monthly seizure frequency	38.30 (78.27)	9.41 (28.74)	-	-	Active TLE group > nonactive TLE group	0.0977
Number of medications	1.82 (1.01)	1.53 (0.80)	-	-	Active TLE group > nonactive TLE group	0.3679
Result of MRI (positive/negative)	7/10	7/10	0/11	0.0246*	Active TLE group > healthy control	0.0233*
					Nonactive TLE group > healthy control	0.0233*
Comorbidity of epilepsy	-	-	-	-	-	-
Cognitive impairment	5/17	3/17	-	-	-	-
Depression	2/17	0/17	-	-	-	-
Anxiety	1/17	0/17	-	-	-	-
Vaccination	15/2	16/1	11/0	0.7760		
(Accept/Refuse)	14	15	11	-		
The triple-vaxxed	1	1	0	-		
The double-vaxxed	-	-	-	-		

### Inflammatory factors

The results in Table 2 indicate that levels of interferon-γ (IFN-γ) were significantly elevated in the active TLE group compared to both the nonactive TLE group and HCs (*P* < 0.05). Similarly, levels of IL-1β, TNF-α, IL-5, and IL-10 were significantly higher in the active TLE group than in the nonactive TLE group (*P* < 0.05). When compared to HCs, the levels of IL-1β (*P* = 0.0573) and TNF-α (*P* = 0.0633) in the active TLE group were also elevated, although these differences did not reach statistical significance. No significant group differences were observed for IL-2 and IL-8 among the three groups.

**Table 2.** Serum inflammatory factors of study participants. Data are means (SD). *P* values are for one way-ANOVA or Kruskal-Wallis test. Statistical significance between groups is indicated by bold styling and asterisk (*P < 0.05).

	Post–COVID-19 active TLE (*n* = 17)	Post–COVID-19 nonactive TLE (*n* = 17)	Healthy control (*n* = 11)	*P* value	Contrasts	*P* value
**Proinflammatory**						
					Active TLE group > nonactive TLE group	0.0143*
IL-1β	2.07 (1.73)	0.97 (0.30)	1.07 (0.30)	0.0109*	Active TLE group > HC	0.0573
					Nonactive TLE group < HC	0.9687
TNF-α	3.68 (3.76)	1.43 (0.75)	1.55 (0.33)	0.0159*	Active TLE group > nonactive TLE group	0.0223*
					Active TLE group > HC	0.0633
					Nonactive TLE group < HC	0.9909
					Active TLE group > nonactive TLE group	0.0171*
IFN-γ	3.79 (3.48)	1.64 (0.46)	1.72 (0.35)	0.0113*	Active TLE group > HC	0.0473*
					Nonactive TLE group < HC	0.9952
					Active TLE group > nonactive TLE group	0.0434*
IL-5	2.16 (1.86)	1.15 (0.29)	1.39 (0.25)	0.0466*	Active TLE group > HC	0.2295
					Nonactive TLE group < HC	0.8517
IL-2	1.90 (1.72)	1.51 (0.48)	1.46 (0.17)	0.4871	-	-
IL-8	23.01 (40.71)	37.64 (109.90)	29.95 (34.80)	0.8487	-	-
**Anti-inflammatory**						
					Active TLE group> nonactive TLE group	0.0481*
IL-10	2.71 (2.55)	1.37 (0.37)	1.41 (0.15)	0.0346*	Active TLE group> HC	0.0999
					Nonactive TLE group< HC	0.9981

### TSPO changes in patients with post–COVID-19 active TLE or nonactive TLE versus HCs

**Fig. 1.**
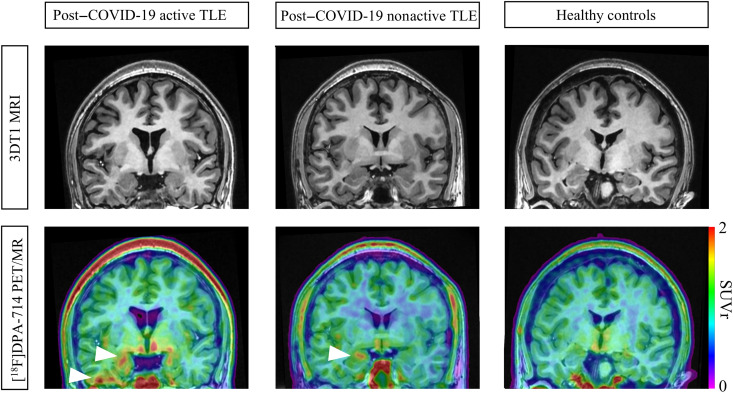
[^18^F]DPA-714 PET/MR of patients with TLE and HCs. 3DT1-MRI (top), [^18^F]DPA-714 PET/MR (bottom) images of three representative participants [one case of a patient with post–COVID-19 active TLE (left), one case of a patient with post–COVID-19 nonactive TLE (middle), and one case of an HC (right)]. Focal uptake in the hippocampus/temporal lobe of the affected hemisphere is marked with an arrow.

**Fig. 2.**
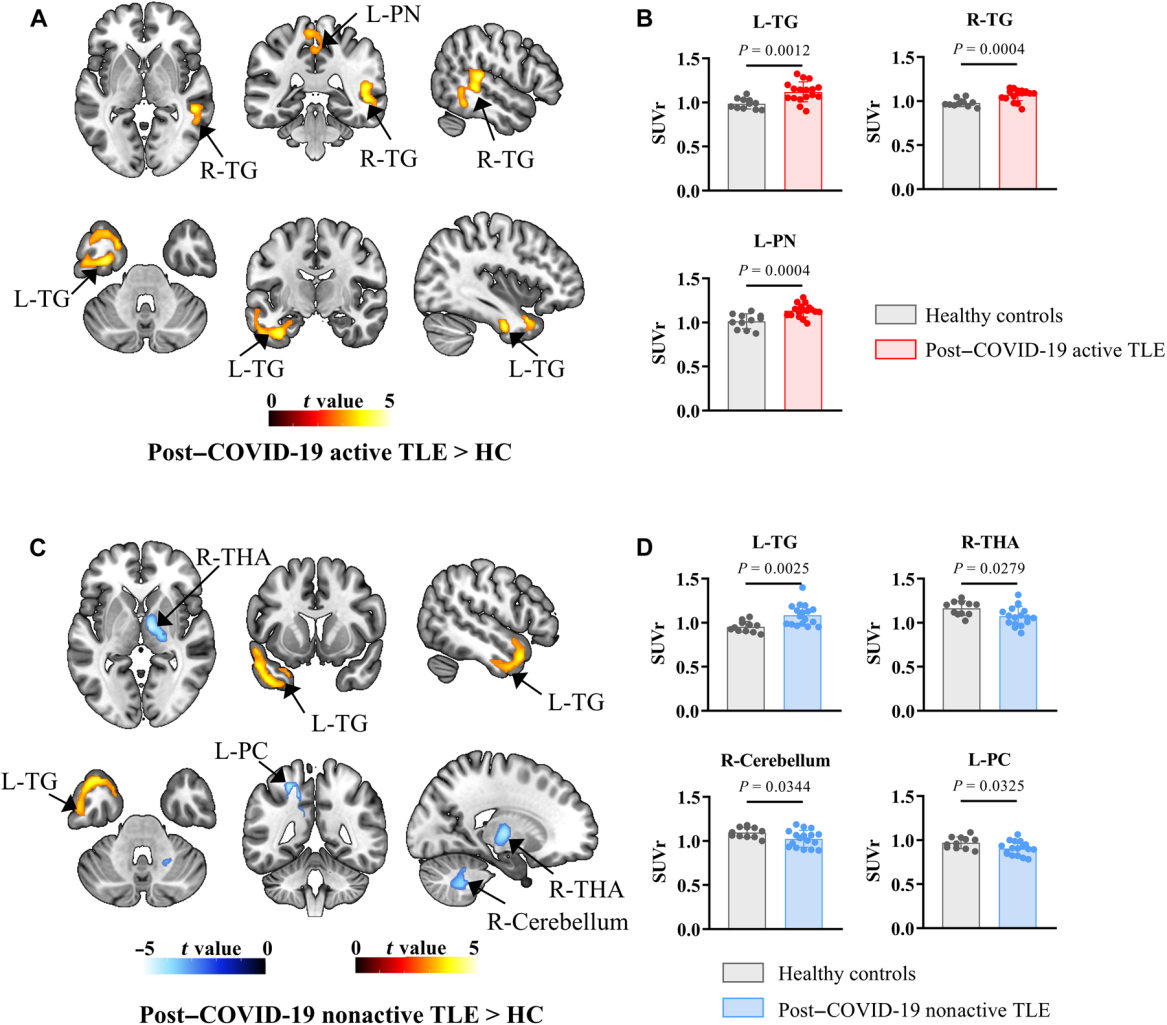
[^18^F]DPA-714 uptake (post–COVID-19 active TLE or post–COVID-19 nonactive TLE versus HC). (**A**) Significant clusters of patients with post–COVID-19 active TLE versus HC and (**C**) patients with post–COVID-19 nonactive TLE versus HC. (**B** and **D**) Visualization of the mean [^18^F]DPA-714 SUVr extracted from subportions of the cluster statistically significant in (A) and (C). L, left; L-PC, left posterior cingulum; L-PN, left precuneus; L-TG, left temporal gyrus; R, right; R-TG, right temporal gyrus; R-THA, right thalamus.

Figure 1 displays images of three representative participants: those with post–COVID-19 active TLE, those with post–COVID-19 nonactive TLE, and controls. Visual assessment revealed that patients with post–COVID-19 nonactive TLE showed a restricted increase [^18^F]DPA-714 uptake in the hippocampus ipsilateral to the epileptogenic focus compared to controls. Conversely, patients with post–COVID-19 active TLE exhibited a more extensive increase [^18^F]DPA-714 uptake, affecting the hippocampus and the temporal lobe ipsilateral to the epileptogenic focus. Voxel-wise statistical parametric mapping (SPM) analysis revealed that, compared with HCs, patients with nonactive TLE exhibited increased [^18^F]DPA-714 uptake in the left temporal gyrus and decreased uptake in the right thalamus, the right cerebellum, and the left posterior cingulum. Patients with active TLE displayed increased [^18^F]DPA-714 uptake in both the bilateral temporal gyrus and the left precuneus, with no brain regions showing decreased uptake (*P* < 0.001 uncorrected, clusters of ≥200 voxels; refer to Table 3 and Fig. 2).

**Table 3.** Results of SPM analysis of [^18^F]DPA-714 uptake (post–COVID-19 active TLE or post–COVID-19 nonactive TLE versus HCs). n.s., not significant.

	Anatomical region	Brodmann area	Coordinates (*x*, *y*, *z*) (mm)			Peak-level *T*	Peak-level *Z*	Cluster-level *K*_E_
Post–COVID-19 active TLE > HC	R Superior temporal gyrus	-	50	−32	10	4.568293571	3.878611647	639
	R Middle temporal gyrus	-	50	−36	0	4.284055233	3.692393074	-
	R Middle temporal gyrus	-	54	−48	−4	3.773635387	3.338886778	-
	L Fusiform	-	−34	−10	−34	4.44302845	3.79746029	1268
	L Temporal pole	-	−34	10	−28	3.973747253	3.480461034	-
	L Inferior temporal gyrus	-	−56	−14	−28	3.87105155	3.408291907	-
	L Median cingulum	Brodmann area 5	−2	−42	50	3.828528881	3.378109873	456
	L Paracentral lobule	-	−4	−34	64	3.575965643	3.195200913	-
	L Precuneus	-	−8	−42	58	3.461898804	3.110535576	-
Post–COVID-19 active TLE < HC	n.s.	-	-	-	-	-	-	-
Post–COVID-19 nonactive > HC	L Superior temporal gyrus	-	−60	0	−8	−4.628823	−3.771751	903
	L Inferior temporal gyrus	-	−40	12	−38	−4.240885	−3.539751	-
	L Temporal pole	-	−42	16	−30	−4.203695	−3.516741	-
Post–COVID-19 nonactive < HC	White matter	-	8	−4	2	−4.672254	−3.796837	518
	R Thalamus	-	20	−16	4	−4.343091	−3.602282	-
	L Inferior parietal	-	−24	−46	52	−4.453716	−3.668817	224
	White matter	-	−16	−44	44	−4.271926	−3.558851	-
	L Posterior cingulum	-	−12	−48	28	−4.095364	−3.448929	-
	R Cerebellum	-	22	−54	−44	−4.187469	−3.506659	308
	R Cerebellum	-	12	−52	−28	−3.88668	−3.314932	-
	R Cerebellum	-	22	−52	−32	−3.286828	−2.904133	-

### Gray matter and TSPO changes in patients with post–COVID-19 active TLE versus nonactive TLE

**Fig. 3.**
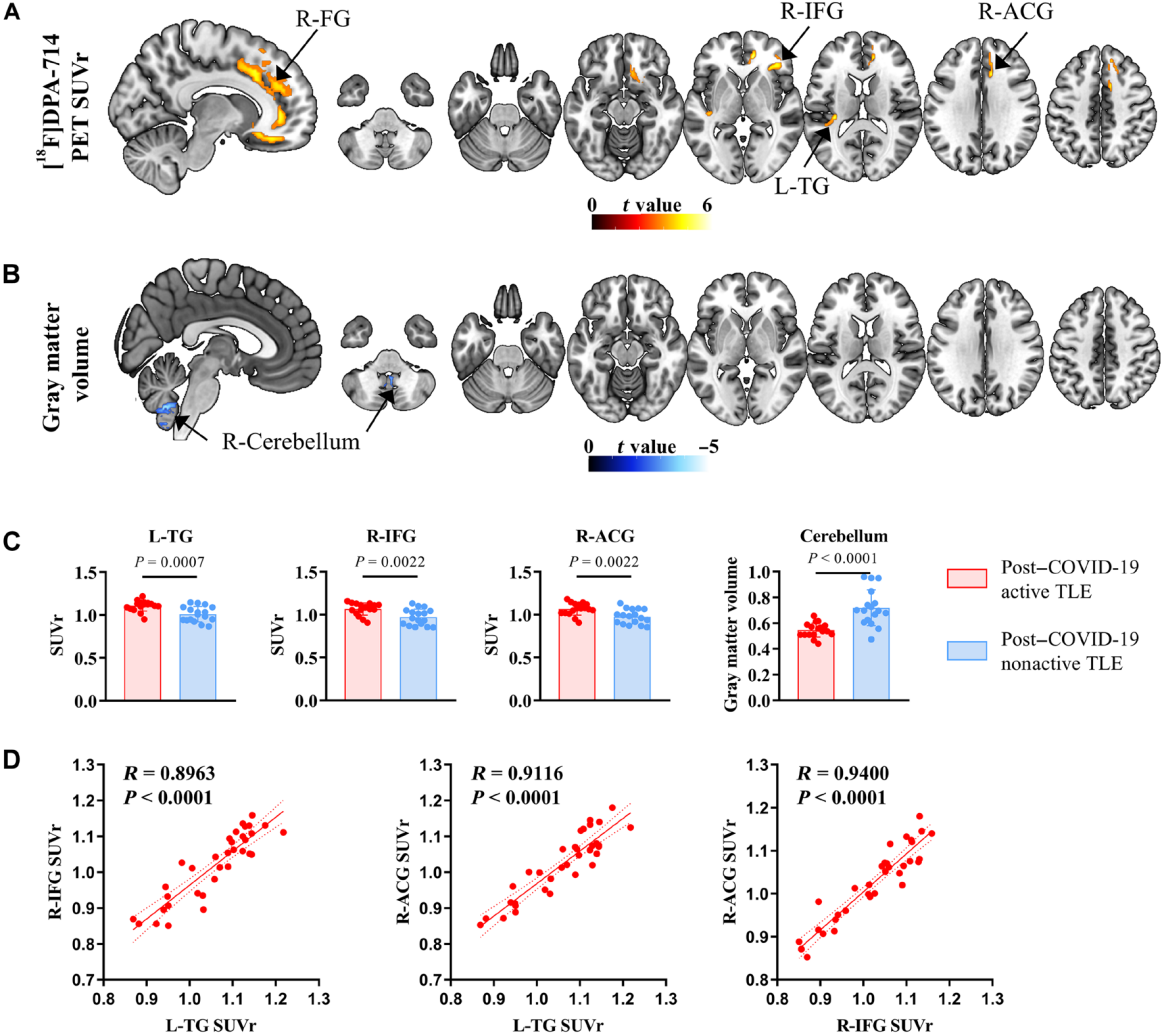
[^18^F]DPA-714 uptake (post–COVID-19 active TLE versus post–COVID-19 nonactive TLE). Significant clusters of (**A**) [^18^F]DPA-714 uptake and (**B**) GM volume of patients with post–COVID-19 active TLE versus patients with post–COVID-19 nonactive TLE versus HC. (**C**) Visualization of the mean [^18^F]DPA-714 SUVr or GM volume extracted from subportions of the cluster statistically significant in (A) and (B). (**D**) Stronger L-TG, R-IFG, and R-ACG correlation in [^18^F]DPA-714 uptake. Solid lines indicate linear fits, and dashed lines indicate standard error of the means. R-ACG, right anterior cingulum; R-FG, right frontal gyrus; R-IFG, right frontal inferior gyrus.

Compared to patients with nonactive TLE, patients with active TLE exhibited increased [^18^F]DPA-714 uptake in the left temporal gyrus, right inferior frontal gyrus, and right cingulum, but decreased gray matter (GM) volume in the cerebellum (*P* < 0.001 uncorrected, clusters of ≥200 voxels; refer to Table 4 and Fig. 3, A to C). Furthermore, a correlation was identified between the SUVr values in the brain regions associated with elevated [^18^F]DPA-714 uptake, specifically in the left temporal gyrus, right inferior frontal gyrus, and right cingulum (*P* < 0.001; Fig. 3D).

**Table 4.** Results of SPM analysis of [^18^F]DPA-714 uptake (post–COVID-19 active TLE versus post–COVID-19 nonactive TLE).

	Anatomical region	Brodmann area	Coordinates (*x*, *y*, *z*) (mm)			Peak-level *T*	Peak-level *Z*	Cluster-level *K*_E_
Post–COVID-19 active TLE > post–COVID-19 nonactive TLE	L Superior temporal gyrus	-	−40	−26	4	5.932261	4.836618	219
	L Rolandic operculum	Brodmann area 13	−32	−30	16	5.319002	4.469132	-
	L Rolandic operculum	-	−40	−36	22	4.831261	4.155683	-
	R Anterior cingulum	-	12	40	2	5.597796	4.639752	708
	R Median cingulum	-	12	20	34	5.518544	4.591865	-
	R Anterior cingulum	Brodmann area 32	12	36	24	5.106804	4.335147	-
	R Inferior frontal gyrus	-	38	32	−2	4.938747	4.226437	208
	R Inferior frontal gyrus							
	R Insula		34	26	8	4.651162	4.034951	-
	R Inferior frontal gyrus	Brodmann area 47	32	28	−10	4.516761	3.943051	-
Post–COVID-19 active TLE < post–COVID-19 nonactive TLE	n.s.	-	-	-	-	-	-	-

### Correlations with PET imaging and inflammatory factors

**Fig. 4.**
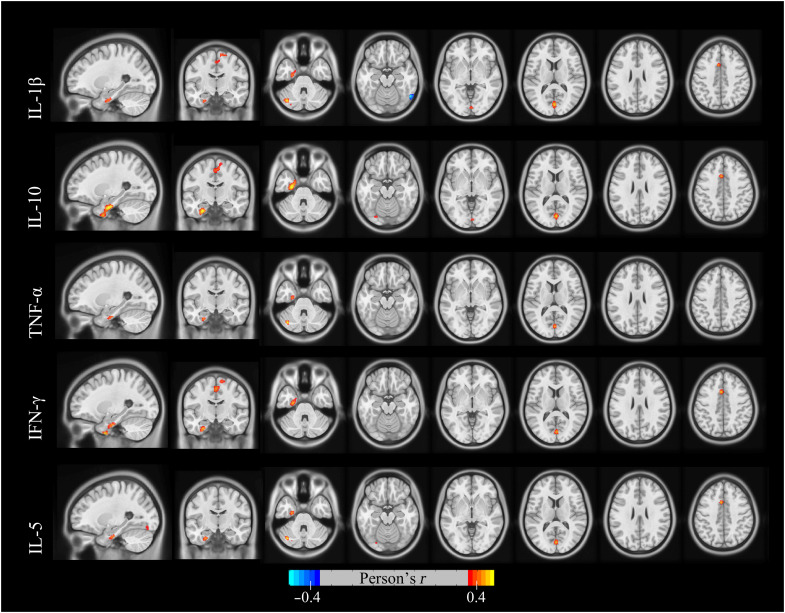
Associations of inflammatory factors with [^18^F]DPA-714 PET at the voxel level. Associations were tested using voxel-wise, univariate, independent, linear regression models with age, sex, and years of education as covariates. The inflammatory factors, including IL-1β, IL-10, and IFN-γ, exhibited the strongest association with [^18^F]DPA-714 PET in the left temporal gyrus. Statistical significance was set at *P* < 0.05, uncorrected for multiple comparisons, with a cluster size of *K*_E_ > 50 voxels.

We further investigated the voxel-wise associations between [^18^F]DPA-714 PET imaging and each of the serum inflammatory factors and found that IL-1β, IL-10, and IFN-γ were the inflammatory factors that had the strongest association with [^18^F]DPA-714 uptake in left temporal gyrus (Fig. 4). A similar relationship was found in IL-5 and TNF-α, although somewhat weaker.

### Cellular origin of TSPO expression in hippocampal tissue

To verify the predominant cellular sources of TSPO expression, we performed multiplex immunohistochemistry staining on hippocampal specimens from patients with post–COVID-19 active TLE and post–COVID-19 nonactive TLE, as well as from non-epileptic postmortem controls. Colocalization analysis demonstrated relatively high overlap between TSPO and ionized calcium-binding adapter molecule 1 (IBA1), a microglia marker (fig. S1). In contrast, TSPO showed limited coexpression with glial fibrillary acidic protein (GFAP), an astrocytic marker (fig. S2).

## DISCUSSION

This study provides previously unidentified evidence of elevated neuroinflammation in patients with post–COVID-19 active epilepsy. Compared to individuals with post–COVID-19 nonactive epilepsy and HCs, patients with active TLE exhibited elevated levels of inflammatory markers (IL-1β, IFN-γ, TNF-α, IL-5, and IL-10). In vivo imaging with TSPO PET indicated a widespread increase in [^18^F]DPA-714 uptake that extended beyond the epileptogenic foci, despite structural MR imaging (MRI) showing no abnormalities. Notably, plasma levels of IL-1β, IL-10, and IFN-γ exhibited the strongest correlation with TSPO PET in areas known for early microglia accumulation, namely, in the medial temporal lobe. These findings implied that both central and systemic inflammation might contribute to epileptogenesis in TLE following COVID-19, thereby suggesting potential therapeutic strategies targeting inflammation in TLE.

Severe acute respiratory syndrome coronavirus 2 (SARS-CoV-2) can access the CNS through two primary pathways. First, it can be transported to the brain via retrograde axonal transport through the cribriform plate in the nasal cavity (*30*). Alternatively, SARS-CoV-2 can spread through the systemic circulation, resulting in the excessive release of inflammatory cytokines, known as the “cytokine storm” (*31*, *32*). The abrupt surge of proinflammatory cytokines during this systemic immune response can disrupt the integrity of the blood-brain barrier (BBB). This disruption compromises the protective functions of endothelial cells, pericytes, and astrocytes, thereby facilitating the infiltration of peripheral immune cells into the CNS. Once within the brain parenchyma, these immune cells may exert cytotoxic effects on resident neural cells, including neurons, potentially contributing to neuroinflammation and neuronal injury (*33*–*36*). Our findings revealed increased levels of inflammatory factors in blood samples from patients with epilepsy post–COVID-19, confirming the presence of a systemic immune-inflammatory response. Simultaneously, TSPO PET captured elevated [^18^F]DPA-714 uptake throughout the brains of patients with TLE, affirming the central inflammatory reaction. Notably, this is the first study to evaluate the systemic and central immune-inflammatory responses in vivo in a single cohort of patients with epilepsy post–COVID-19. Not only that, it also confirms the coexistence of a “systemic-central” immune response in patients with TLE after COVID-19 infection, although the specific regulatory and interactive mechanisms require further clarification.

Among patients with post–COVID-19 nonactive TLE, elevated [^18^F]DPA-714 uptake was observed compared to controls, indicating the presence of consistent neuroinflammation, as reported in previous studies (*9*, *15*). Conversely, reduced [^18^F]DPA-714 uptake was noted in the thalamic and cerebellum contralateral to the epileptogenic foci, the underlying mechanism of which remains inadequately understood. Following a comprehensive exploration of glial cells and neuroinflammation, researchers have unveiled the complexity of microglial inflammatory states, which no longer fit into simple proinflammatory or anti-inflammatory categories; instead, they display dynamic changes and assume diverse roles varying by brain region, developmental stage, and external stimuli (*37*). It is postulated that the observed reduction in [^18^F]DPA-714 uptake in contralateral thalamic and cerebellum may be linked to distinct microglial states in different subpopulations across various brain regions. This hypothesis requires validation through preclinical experiments in the future. Considering the interacted structural, functional, and metabolic connectome among the temporal lobe, thalamus, and cerebellum in the TLE network, it is further inferred that microglia with distinctive functional roles in the contralateral thalamus and cerebellum may participate in the complex regulation of the TLE network and in maintaining functional brain homeostasis (*38*–*42*).

Compared with both post–COVID-19 nonactive TLE group and HCs, plasmatic inflammatory factors including IL-1β, IL-10, TNF-α, IFN-γ, and IL-5 were significantly elevated in the post–COVID-19 active TLE group. In addition, there was a more widespread elevation of [^18^F]DPA-714 uptake in brain regions, including the ipsilateral temporal, the contralateral anterior cingulate gyrus, and the frontal lobe. Furthermore, the significant positive correlations in the SUVr among these brain regions suggest the presence of activated microglia throughout the brain, outside the epileptogenic foci area, and the persistence of neuroinflammation for an extended period, exacerbating seizure frequency and intensity in patients with post–COVID-19 active TLE. In essence, the inflammation of central neuroglial cells contributes to the progression of epilepsy. It has been demonstrated in some clinical cases, such as that of a 2-year-old boy with ultrarefractory focal status epilepticus persistent in the setting of acute COVID-19 and multisystem inflammatory syndrome in children, who showed reduced seizure frequency after receiving high-dose intravenous methylprednisolone following multiple antiseizure medication (ASM) loading failures and continued midazolam infusion dependence (*43*). Therefore, the clinical application of TSPO PET evaluation and immunotherapeutic intervention holds promise for patients with post–COVID-19 active epilepsy, especially those with refractory epilepsy.

Previous investigations have indicated microglial activation related to the frequency of seizures, suggesting neuroinflammation as a key determinant of the intrinsic severity of epilepsy (*44*, *45*). Voxel-wise correlation analysis revealed that IL-1β, IL-10, and IFN-γ were the plasma biomarkers demonstrating the strongest correlation with TSPO PET in regions known for early microglia accumulation, specifically the medial temporal lobe. Therefore, in patients with increased seizure frequency TLE, elevated blood levels of these inflammatory markers indicate a strong indication for further assessment of cerebral TSPO PET and consideration of immunotherapy. IL-1β is a crucial contributor to epilepsy. The activation of inflammasome will lead to the autoactivation of caspase-1 and subsequent cleavage of proIL-1β, which are key sources of inflammatory manifestations (*16*, *46*). Clinical studies assert that the levels of IL-1β during the interictal period in epileptics are higher than that in HCs, and targeting IL-1 receptor signaling is effective in controlling drug-resistant epilepsy (*47*–*49*). IL-10 is an anti-inflammatory cytokine predominantly expressed by astrocytes and microglia in the CNS, exerting protective effects against inflammation and apoptosis (*50*). In a rat model of pilocarpine-induced status epilepticus, a notable up-regulation of IL-10 expression in the hippocampus has been documented (*51*). Furthermore, in patients with autoimmune epilepsy, elevated serum IL-10 is linked to high levels of anti-glutamic acid decarboxylase (GAD) autoantibodies (*52*), highlighting its role in inflammation and drug resistance development in epilepsy. IFN-γ is a recently identified epileptogenic factor, with limited available study data (*53*). Its elevation in serum specimens from epileptic patients suggests a potential role in epilepsy progression by inducing the release of inflammatory mediators, such as TNF-α and IL-1β, and disrupting the BBB (*54*). However, the primary experiments about immune inflammation in epilepsy are preclinical studies. Our research provides pivotal in vivo evidence from clinical patients, declaring that ascending plasmatic inflammatory factors like IL-1β, IL-10, and IFN-γ may drive neuroinflammation and facilitate epileptogenesis. Targeting inflammatory factors such as IL-1β, IL-10, and IFN-γ represents a promising strategy for immunoinflammatory treatments for epilepsy.

Our research not only evaluated inflammatory responses using TSPO PET but also analyzed structural changes via MRI. Compared with structural MRI, in-vivo TSPO PET images have a prominent advantage in sensitively and stably depicting TLE neuroinflammation, making them more valuable in clinical evaluation. TSPO PET could exhibit pathological functional alteration early, which is conducive to exploring the etiology and finding precipitating factors of disease progression in depth. Nevertheless, structural lesions displayed by MRI always lag behind functional changes and typically appear as late clinical manifestations. Consequently, TSPO PET can be a valuable tool for early clinical diagnosis, continuous monitoring, and assessment of therapeutic efficacy concerning neuroinflammation in patients with TLE.

The study does have several limitations. First, the relatively small sample size and single-center design may limit the generalizability of our findings. Future research should include larger, multicenter cohorts to validate these results and enable subgroup analyses based on comorbidities, seizure types, and vaccination status. Second, both patients and HCs have experienced COVID-19 infection. Ideally, participants without any COVID-19 exposure would result in more comprehensive and convincing findings. However, given the current epidemic situation in China, nearly everyone has been infected with COVID-19 at least once. Third, we have found a positive correlation between TSPO radiotracer uptake and plasmatic inflammatory cytokines; further evidence is needed to evaluate how TSPO radiotracer uptake and these inflammatory factors change in response to immune intervention therapies, and this will be a focus of our future research. Fourth, the up-regulation of TSPO expression is not entirely specific to glial cells. Although TSPO is typically expressed in microglia in neuropsychiatric diseases, the next most common cellular expression occurs in endothelial cells (*55*, *56*). However, the content of endothelial cells is unlikely to fully account for the findings, as their spatial extent is limited to surrounding blood vessels, and the vascular contribution to PET is less than 5% of the input signal in the brain (*57*, *58*). Fifth, the high-affinity binding genotype of TSPO is more prevalent among East Asians and less common in other ethnicities, making the applicability of TSPO radioligand in Caucasians unknown (*27*, *59*, *60*). Last, the association between neuroinflammation and epilepsy is convoluted. Up-regulated neuroinflammation may reduce the threshold of epileptogenesis, whereas recurrent seizures could exacerbate inflammation. Our research could not establish a definitive causal relationship, indicating the need for further in-depth exploration.

In conclusion, our research demonstrates widely distributed neuroinflammation and elevated plasmatic inflammatory cytokines in patients with post–COVID-19 active TLE. In addition, the levels of plasma IL-1β, IL-10, and IFN-γ are closely associated with neuroimmune activation, particularly in the ipsilateral temporal lobe. This suggests that combining plasma inflammatory factors with TSPO PET imaging could be a reliable approach for clinical diagnosis, dynamic monitoring, and assessment of immunotherapeutic efficacy in neuroinflammation associated with TLE.

## MATERIALS AND METHODS

### Study participants

This prospective single-center study was carried out at Xiangya Hospital of Central South University from June 2023 to July 2025, involving patients with a confirmed diagnosis of TLE (*61*) based on seizure symptoms, neurological examination, 3-T MRI, video-electroencephalography, and neuropsychological assessment. The inclusion criteria were as follows: (i) aged between 18 and 70 years, (ii) had a confirmed history of SARS-CoV-2 infection based on reverse transcription polymerase chain reaction test results, and (iii) had no contraindications for PET/MR scanning. Exclusion criteria included the following: (i) the use of immunosuppressive agents following COVID-19 infection, (ii) the presence of established neurological or psychiatric illness other than epilepsy, and (iii) having a low-affinity genotype for TSPO (rs6971 gene) (*55*, *62*). “An increase in seizure severity” was defined as an increase in the frequency or duration of epileptic seizures by twofold or more, or the transformation into drug-resistant epilepsy (*63*), that is, when two tolerated and appropriately selected ASMs fail to control epileptic seizures. Based on the observed increase in seizure severity, patients with TLE were categorized into two groups: those with worsening seizure symptoms after COVID-19 infection (post–COVID-19 active TLE) and those without worsening seizure symptoms after COVID-19 infection (post–COVID-19 nonactive TLE).

In addition, 11 HCs, matched for age and sex with patients with TLE, were recruited from the patients’ families and the local community. The exclusion criteria encompassed neurological or psychiatric disorders, drug addiction, and structural abnormalities detected on MRI scans.

The study was approved by the Ethics Committee of Xiangya Hospital of Central South University (no. 202305120). All the patients signed an informed consent form for the PET examination and the use of medical data.

### Measurement of serum inflammatory factors

Blood samples were collected from all participants at the onset of each PET/MR session. Plasma samples were obtained from both controls and patients with TLE for the assessment of ILs (IL-1β, IL-2, IL-5, IL-8, and IL-10), TNF-α, and tumor-specific IFN-γ. The levels of these cytokines were quantified using enzyme-linked immunosorbent assay kits (*64*).

### PET/MR image acquisition

[^18^F]DPA-714 PET and MRI data were acquired using a PET/MR system (GE HealthCare). PET imaging was conducted in the list mode 30 to 60 min after intravenous injection of 3.7 MBq/kg of [^18^F]DPA-714, following a previously published standardized procedure (*65*, *66*). Concurrently, the following brain MRI scans were obtained: a three-dimensional T1-weighted anatomical scan and a resting-state functional MRI scan. The detailed MRI sequences are shown in table S2. PET images were reconstructed with 256 by 256 pixels, using eight iterations, 18 subsets, and a filter with a full width at half maximum (FWHM) of 2 mm using a point spread function algorithm. Patients were carefully monitored to ensure the absence of seizures within 24 hours before the scans, and participants were instructed to minimize head movement during the scanning procedure as much as possible.

### MRI data preprocessing

Prior to analysis, the image quality was visually inspected, and the origin of each T1-weighted image was set to the anterior commissure. Images of the patients with right-sided lesions were flipped to facilitate analysis. The reoriented images were then spatially normalized to the Montreal Neurological Institute (MNI) space, correcting for intensity variations, and were segmented into tissue compartments using the segment routine of the SPM12 (www.fil.ion.ucl.ac.uk/spm/) (*67*). To account for the impact of spatial normalization on volumetric data, a modulation process was applied to the GM probability maps. Last, the modulated GM probability maps were smoothed with an 8-mm FWHM Gaussian kernel, resulting in a voxel size of 1.5 mm by 1.5 mm by 1.5 mm.

### PET data preprocessing

PET image processing was conducted using the SPM12 toolkit on a MATLAB platform (R2018b). Initially, individual scans of [^18^F]DPA-714 underwent motion correction. Lesions on the right were flipped to the left for analysis purposes. Next, PET images were automatically aligned with the T1-weighted image space, while the T1-weighted images were registered both linearly and nonlinearly to the MNI space and resliced to 2 mm by 2 mm by 2 mm. Normalized PET images were spatially smoothed with an FWHM Gaussian kernel of 8 mm to accommodate individual differences in anatomy. SUV was corrected for body weight and radiotracer decay, and a histogram-based intensity normalization method was applied to eliminate the effects of physiological nuisances. The SUVr was then calculated using the cerebellar cortex as a reference region (*66*, *68*).

### PET and MRI data analysis

The voxel-wise group comparisons of GM or SUVr were performed using the two-sample *t* test model of SPM12. Covariates including sex, age at PET, and years of education were used for comparing patients with TLE with HCs, while additional covariates such as age at seizure onset, seizure frequency, and number of drugs were incorporated for comparisons among different TLE subgroups. The significance threshold was set at *P* < 0.001 (uncorrected), with cluster significance thresholds at 200 contiguous voxels.

The cluster-level statistics of group comparison for GM and SUVr, including the number of voxels, and MNI coordinates for the peak voxels, were calculated based on SPM12, and corresponding anatomical labels were obtained using xjView toolbox (www.alivelearn.net/xjview).

Regions of interest (ROIs) demonstrating significant intergroup differences were identified, including the cerebellum for GM, and areas such as the left temporal gyrus, left precuneus, right temporal gyrus, right thalamus, right inferior frontal gyrus, right cingulum, and right cerebellum for SUVr, which were generated by intersecting the subregions of the automated anatomical labeling atlas (*69*) with the significant SPM clusters. Subsequently, the GM and SUVr values within these ROIs were computed for visualization and correlational analyses. The association between SUVr across different cluster parcellates was assessed using Spearman’s coefficient.

### Voxel-wise directed correlations between PET imaging and inflammatory factors

To estimate the relationship between PET imaging and various inflammatory factors (including IL-1β, IL-5, IL-10, TNF-α, and IFN-γ), we used the DPABI toolbox (http://rfmri.org/dpabi), which uses a general linear model to compute voxel-wise correlations. Age, sex, and years of education were controlled for as covariates in the analysis. The DPABI Viewer was used to visualize the results (*70*).

### Statistical analysis

Whole-brain voxel-wise analyses were conducted using SPM12, including group comparison of MRI and TSPO PET data. We used a two-sample *t* test and multiple regression designs. Results with a height threshold of *P* < 0.001 (uncorrected) and cluster significance thresholds at 200 contiguous voxels were considered significant.

For clinical and biomarker comparisons, as well as semiquantitative analyses of GM and regional SUVr values derived from MRI and PET, statistical analyses were performed using GraphPad Prism 8.0 (GraphPad Software). Comparative analysis of patient characteristics involved implementing χ^2^ tests for categorical variables and one-way analysis of variance (ANOVA) for continuous variables, with significance set at *P* < 0.05. For correlations between PET imaging and inflammatory factors, DPABI generated results with the significance level set at *P* < 0.05 (uncorrected) and cluster significance thresholds at 50 contiguous voxels.
