# Supplementary material for: Neuroimmune activation in temporal lobe epilepsy patients with worsening seizure following the COVID-19 pandemic: A [18F]DPA-714 PET/MR study
Source: Sci Adv. 2026 Jan 30;12(5):eadu5874. doi: 10.1126/sciadv.adu5874 (PMC12857733; doi:10.1126/sciadv.adu5874)
Supplement: Supplementary file 1 — Supplementary Text Figs. S1 to S3 Tables S1 to S3 [file sciadv.adu5874_sm.pdf]

Science Advances
AAAS

Supplementary Materials for

Neuroimmune activation in temporal lobe epilepsy patients with worsening seizure following the COVID-19 pandemic: A [^{18}F]DPA-714 PET/MR study

Ling Xiao *et al.*

Corresponding author: Yongxiang Tang, 405035@csu.edu.cn; Shuo Hu, hushuo2018@163.com;
Li Feng, fenglihx@163.com

Sci. Adv. **12**, eadu5874 (2026)
DOI: 10.1126/sciadv.adu5874

This PDF file includes:

Supplementary Text
Figs. S1 to S3
Tables S1 to S3

Supplementary Text

Supplementary Results

TSPO expression in hippocampal tissues from TLE patients before and after COVID-19 infection

To investigate TSPO expression patterns, we performed multiplex immunohistochemistry (mIHC) staining on hippocampal tissues obtained from three groups: (1) drug-resistant TLE patients who underwent anterior temporal lobectomy before the COVID-19 pandemic, (2) drug-resistant TLE patients who underwent surgery after confirmed COVID-19 infection, and (3) postmortem non-epileptic controls. As shown in fig. S3, TSPO immunoreactivity was markedly elevated in hippocampal specimens from post-COVID-19 TLE patients compared with both pre-COVID-19 TLE patients and non-epileptic controls.

Supplementary Methods

Human hippocampal specimens

Human hippocampal tissues were obtained from the archives of the Department of Pathology, Xiangya Hospital, Central South University. A total of 12 hippocampal specimens were collected from patients undergoing anterior temporal lobectomy for drug-resistant epilepsy, including six pre-COVID-19 resections and six post-COVID-19 resections (three post-COVID-19 active and three post-COVID-19 non-active TLE patients). Additionally, six postmortem hippocampal samples from non epileptic individuals without a history of seizures or other neurological diseases were included as controls. All procedures were approved by the Institutional Ethics Committee of Xiangya Hospital and conducted in accordance with the Declaration of Helsinki. Clinical characteristics of the included cases are summarized in table S3.

Multiplex immunohistochemistry

Multiplex IHC staining of hippocampal sections (10 μm) from surgical and postmortem specimens was performed using tyramide signal amplification (TSA) Plus Fluorescence Kits (PerkinElmer, USA) combined with IHC (TSA-IHC). Various primary antibodies, including rabbit anti-TSPO (1:2000, Abcam), rabbit anti-Iba1 (1:5000, Servicebio), and rabbit anti-GFAP (1:5000, Servicebio), were sequentially applied at 37°C for 1 hour, followed by horseradish peroxidase-conjugated secondary antibody incubation and TSA. The slides were microwave heat-treated after each TSA operation. The nuclei were counterstained with DAPI and images were acquired using fluorescence microscope (Nikon, Tokyo, Japan).

Supplementary Figures

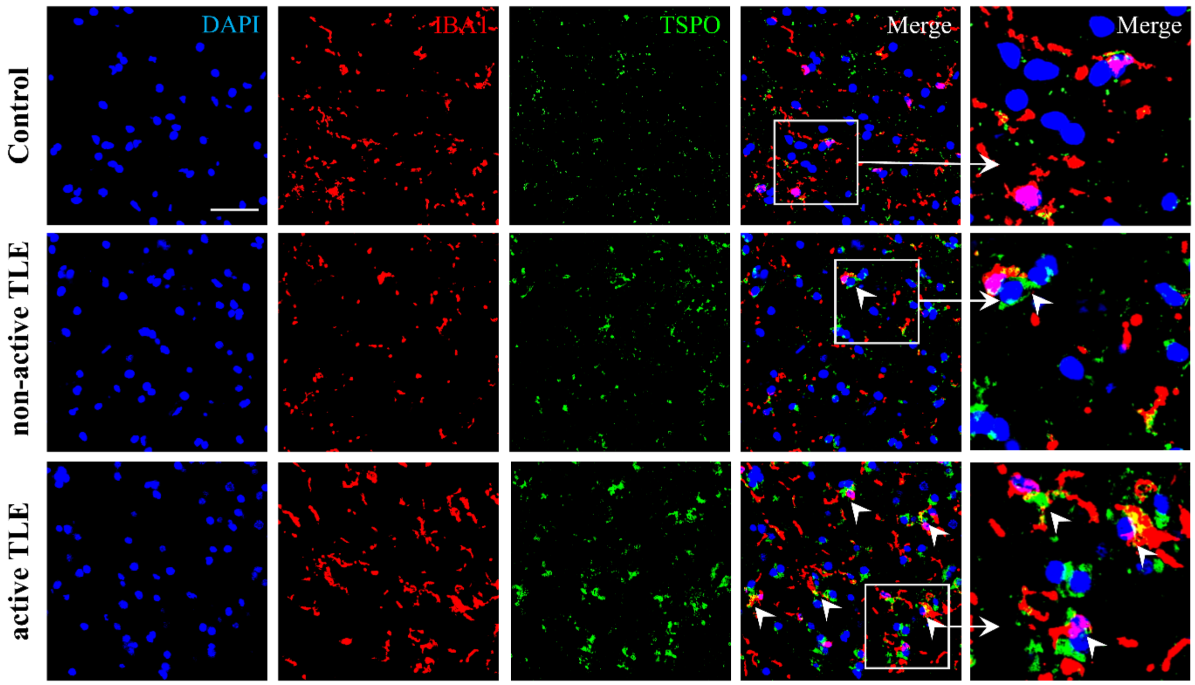

Fig. S1. Co-localization of TSPO with microglial marker IBA1 in hippocampal tissues. Representative mIHC images showing TSPO (green) and IBA1 (red) expression in hippocampal sections from three groups: post-COVID-19 active TLE patients, post-COVID-19 non-active TLE patients, and non-epileptic postmortem controls. Nuclei were counterstained with DAPI (blue). Colocalization analyses demonstrated relatively high overlap between TSPO and IBA1 signals. Arrowheads indicate areas of TSPO–IBA1 co-localization. Scale bar = 50 µm.

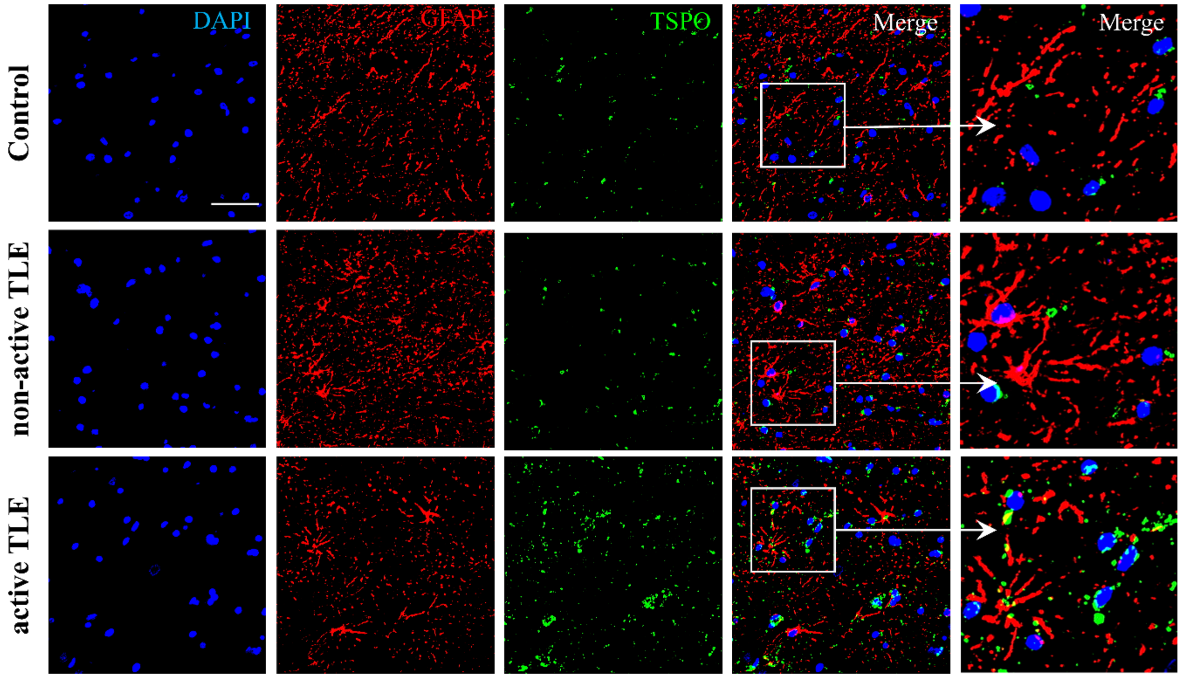

Fig. S2. Co-localization of TSPO with astrocytic marker GFAP in hippocampal tissues. Representative mIHC images showing TSPO (green) and GFAP (red) expression in hippocampal sections from three groups: post-COVID-19 active TLE patients, post-COVID-19 non-active TLE patients, and non-epileptic postmortem controls. Nuclei were counterstained with DAPI (blue). TSPO and GFAP signals exhibited limited colocalization. Scale bar = 50 μm.

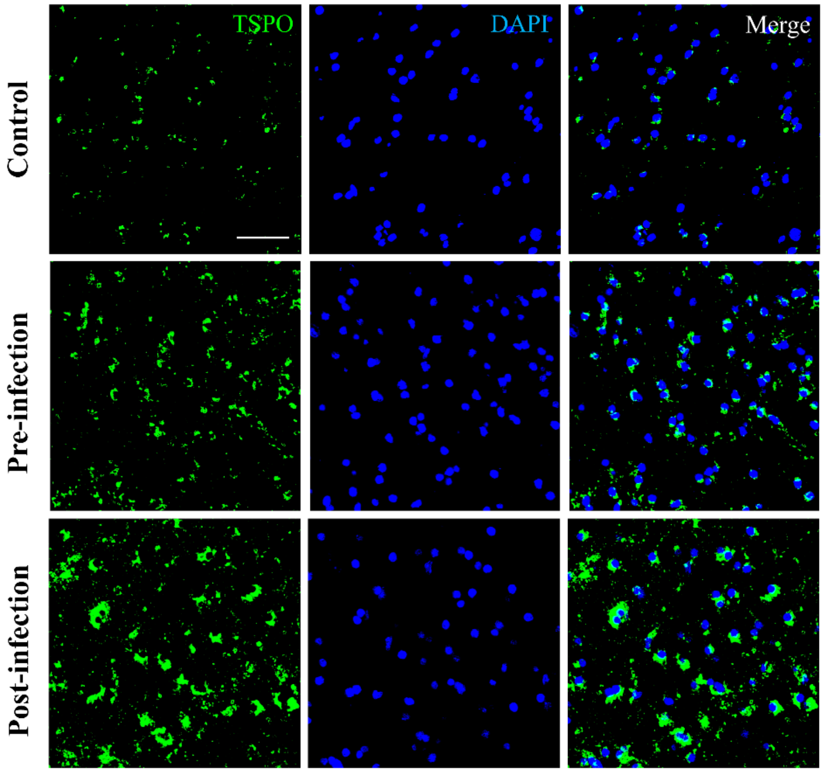

Fig. S3. TSPO expression in hippocampal tissues from pre- and post-COVID-19 TLE patients and controls. Representative mIHC images of TSPO (green) in hippocampal sections from three groups: post-COVID-19 TLE patients, pre-COVID-19 TLE patients, and postmortem non-epileptic controls. Nuclei were counterstained with DAPI (blue). Compared with both pre-COVID-19 TLE patients and controls, hippocampal tissues from post-COVID-19 TLE patients exhibited higher TSPO immunoreactivity. Scale bar =50 μm.

Supplementary Tables

Table S1. Demographic and clinical characteristics of participants.

Participants	Sex	Age	Education years	Seizure onset age	Active TLE/ non-active TLE	Monthly seizure frequency	ASMs	Result of MRI	Comorbidity of epilepsy	Vaccination Condition
Patient No.1	F	35	12	35	active TLE	0.6	OXC	right hippocampus sclerosis	-	accept/ the triple-vaxxed
Patient No.2	F	47	9	42	active TLE	120	LEV	negative	cognitive impairment, depression, anxiety	accept/ the triple-vaxxed
Patient No.3	M	38	16	38	active TLE	300	OXC	negative	-	accept/ the double-vaxxed
Patient No.4	F	20	12	15	active TLE	16	OXC+LTG	negative	-	accept/ the triple-vaxxed
Patient No.5	M	18	12	13	active TLE	16	LCM+ OXC+VPN+PER	negative	cognitive impairment	refuse
Patient No.6	M	62	9	12	active TLE	3	OXC	left hippocampus swelled and left temporal horn of lateral ventricle atrophied	cognitive impairment	refuse
Patient No.7	M	43	9	40	active TLE	1	VPA+OXC	cerebromalacia in left temporal lobe	-	accept/ the triple-vaxxed

Patient No.8	M	58	9	28	active TLE	5	OXC+LEV	left hippocampus sclerosis	cognitive impairment	accept/ the triple-vaxxed
Patient No.9	F	56	9	53	active TLE	120	LCM+LEV	negative	depression	accept/ the triple-vaxxed
Patient No.10	M	36	16	36	active TLE	1	OXC	negative	-	accept/ the triple-vaxxed
Patient No.11	M	45	9	15	active TLE	60	PB+CBZ+VPA+LEV	right hippocampus sclerosis	-	accept/ the triple-vaxxed
Patient No.12	M	47	16	47	active TLE	0.5	LEV+LCM+PER	left hippocampus swelled	-	accept/ the triple-vaxxed
Patient No.13	F	29	12	21	active TLE	4	LEV+OXC	negative	-	accept/ the triple-vaxxed
Patient No.14	M	30	12	28	active TLE	0.5	OXC	right temporal angle is slightly wider	-	accept/ the triple-vaxxed
Patient No.15	F	48	6	46	active TLE	1	OXC	negative	cognitive impairment	accept/ the triple-vaxxed
Patient No.16	M	33	12	27	active TLE	0.5	OXC	negative	-	accept/ the triple-vaxxed
Patient No.17	M	42	12	40	active TLE	2	OXC+LCS	negative	-	accept/ the triple-vaxxed
Patient No.18	M	37	16	17	non-active TLE	3	LCM	negative	-	accept/ the triple-vaxxed
Patient No.19	M	40	9	15	non-active TLE	12	VPN	negative	cognitive impairment	accept/ the triple-vaxxed
Patient No.20	F	37	12	20	non-active TLE	3	LEV	negative	-	accept/ the double-vaxxed
Patient No.21	M	34	12	17	non-active TLE	12	LEV+OXC+LCM	white matter hyperintensity in right frontal lobe	-	accept/ the triple-vaxxed

Patient No.22	F	35	9	24	non-active TLE	2	LEV+OXC+TPM	bilateral hippocampus sclerosis	-	refuse
Patient No.23	F	20	12	15	non-active TLE	1	LEV+OXC	negative	-	accept/ the triple-vaxxed
Patient No.24	F	34	9	28	non-active TLE	2	LTG	right hippocampus sclerosis	-	accept/ the triple-vaxxed
Patient No.25	F	22	16	10	non-active TLE	1	LEV	right hippocampus sclerosis	-	accept/ the triple-vaxxed
Patient No.26	F	34	16	22	non-active TLE	120	LEV+OXC	heterotopic gray matter in left cornu occipitale	cognitive impairment	accept/ the triple-vaxxed
Patient No.27	M	33	16	28	non-active TLE	0.17	LCM	negative	-	accept/ the triple-vaxxed
Patient No.28	F	40	12	8	non-active TLE	1	LCM	negative	-	accept/ the triple-vaxxed
Patient No.29	M	38	12	36	non-active TLE	1	LEV+OXC+LCM	negative	-	accept/ the triple-vaxxed
Patient No.30	M	36	12	30	non-active TLE	0.2	LEV	negative	-	accept/ the triple-vaxxed
Patient No.31	F	26	16	18	non-active TLE	0.2	OXC	negative	-	accept/ the triple-vaxxed
Patient No.32	F	18	12	10	non-active TLE	1	LEV+OXC	right temporal angle is slightly wider	cognitive impairment	accept/ the triple-vaxxed
Patient No.33	M	25	16	19	non-active TLE	0.1	LEV	flattened left hippocampal digitations	-	accept/ the triple-vaxxed
Patient No.34	F	34	12	30	non-active TLE	0.25	LCM	negative	-	accept/ the triple-vaxxed

Control No.1	F	25	20	-	-	-	-	negative	-	accept/ the triple-vaxxed
Control No.2	F	51	12	-	-	-	-	negative	-	accept/ the triple-vaxxed
Control No.3	M	53	12	-	-	-	-	negative	-	accept/ the triple-vaxxed
Control No.4	M	26	9	-	-	-	-	negative	-	accept/ the triple-vaxxed
Control No.5	F	24	20	-	-	-	-	negative	-	accept/ the triple-vaxxed
Control No.6	F	27	20	-	-	-	-	negative	-	accept/ the triple-vaxxed
Control No.7	F	28	12	-	-	-	-	negative	-	accept/ the triple-vaxxed
Control No.8	M	50	9	-	-	-	-	negative	-	accept/ the triple-vaxxed
Control No.9	M	40	9	-	-	-	-	negative	-	accept/ the triple-vaxxed
Control No.10	F	34	6	-	-	-	-	negative	-	accept/ the triple-vaxxed
Control No.11	F	40	6	-	-	-	-	negative	-	accept/ the triple-vaxxed

Abbreviations: ASMs, antiseizure medications; F, female; M, male; TLE, temporal lobe epilepsy; OXC, Oxcarbazepine; LEV, Levetiracetam; LTG, Lamotrigine; PER, Perampanel; VPN, Valproate; CBZ, Carbamazepine; LCM, lacosamide; PB, Phenobarbital; TPM, Topiramate.

Table S2.The implementation details of the head MRI for participants.

Sequences	three-dimensional T1-weighted anatomical scan	a resting-state functional magnetic resonance imaging (fMRI) scan
repetition time	8.5 ms	2000 ms
echo time	3.3 ms	60 ms
slice thickness	1.0 mm	3.0 mm
acquisition matrix	256 × 256	64 × 64
field of view	240 × 240 mm^2	240 × 240 mm^2
voxel size	1.0 × 1.0 × 1.0 mm^3	3.0 × 3.0 × 3.0 mm^3

Table S3. Summary of clinical features in patients and controls according to neuropathology.

	Sex	Age of surgery or death	Pre-COVID-19 surgery/post-COVID-19 surgery	Active TLE/ non-active TLE	Duration of epilepsy
TLE-1	F	32	post-COVID-19 surgery	active TLE	6
TLE-2	F	55	post-COVID-19 surgery	active TLE	40
TLE-3	F	51	post-COVID-19 surgery	active TLE	34
TLE-4	M	18	post-COVID-19 surgery	non-active TLE	2
TLE-5	F	44	post-COVID-19 surgery	non-active TLE	35
TLE-6	M	36	post-COVID-19 surgery	non-active TLE	4
TLE-7	F	21	pre-COVID-19 surgery	-	18
TLE-8	F	17	pre-COVID-19 surgery	-	12
TLE-9	M	45	pre-COVID-19 surgery	-	30
TLE-10	M	30	pre-COVID-19 surgery	-	12
TLE-11	F	34	pre-COVID-19 surgery	-	20
TLE-12	M	40	pre-COVID-19 surgery	-	11
PD-1	M	31	-	-	-
PD-2	M	60	-	-	-
PD-3	M	67	-	-	-
PD-4	M	77	-	-	-
PD-5	F	50	-	-	-
PD-6	F	19	-	-	-

Abbreviations: F, female; M, male; PD, postmortem donor; TLE, temporal lobe epilepsy.
